# A Comprehensive Review of the Epidemiology, Pathophysiology, Risk Factors, and Treatment Strategies for Retinoblastoma

**DOI:** 10.3390/diseases13090307

**Published:** 2025-09-19

**Authors:** Alpana Kumari, Sarav Paul Singh, Pankaj Kumar, Suresh Babu Kondaveeti, Vivek Kumar Garg, Rabdeep Kaur, Harpal Singh Buttar, Katrin Sak, Kiran Yadav, Vikas Yadav

**Affiliations:** 1 Department of Optometry, University Institute of Allied Health Sciences, Chandigarh University, Gharuan, Mohali 140413, Punjab, India; alpana.e11438@cumail.in (A.K.); rabdeepkaur13@gmail.com (R.K.); 2 Chicago DLS Inc., Chicago, IL 60652, USA; saravpaul@gmail.com; 3 Department of Biochemistry, U.P. University of Medical Sciences, Saifai, Etawah 206130, Uttar Pradesh, India; pankajmarshal@gmail.com; 4 Department of Biochemistry, Symbiosis Medical College for Women, Symbiosis International (Deemed University), Pune 412115, Maharashtra, India; ksuresh.babu@smcw.siu.edu.in; 5 Department of Medical Lab Sciences, Rayat-Bahra University, Mohali 140104, Punjab, India; 6 Department of Pathology & Laboratory Medicine, School of Medicine, University of Ottawa, Ottawa, ON K1N 6N5, Canada; hsbuttar@bell.net; 7 NGO Praeventio, 50407 Tartu, Estonia; katrin.sak.001@mail.ee; 8 Faculty of Pharmaceutical Sciences, The ICFAI University, Himachal Pradesh 174103, India; kiran.yadav@iuhimachal.edu.in; 9 Department of Translational Medicine, Clinical Research Centre, Skåne University Hospital, Lund University, SE 20213 Malmö, Sweden

**Keywords:** retinoblastoma, pediatric oncology, genetics, risk factors, immunotherapy, chemotherapy, radiotherapy

## Abstract

The retinoblastoma gene (*RB1*), which is located on chromosome 13q14.2, is mutated in retinoblastoma (RB), the most common malignant intraocular tumor in children. About 8000 new cases of retinoblastoma are diagnosed globally each year, accounting for approximately 1 in 17,000 live births. RB is prototypically considered hereditary by nature as thirty to forty percent of cases have autosomal dominant inheritance, and the remaining sixty to seventy percent have non-inherited sporadic inheritance. RB is the most treatable juvenile malignancy, with a high percentage of survival; nevertheless, advanced tumors restrict the amount of globe salvage and are frequently linked to high-risk histological characteristics that indicate spread. Investigating the disease’s molecular causes has also helped to understand its subsequent processes, which has resulted in the identification of biomarkers and relevant targeted treatments. Additionally, advancements in molecular biology techniques facilitated the creation of effective strategies for early disease detection, genetic counseling, and prevention. In the present review, we discuss the risk factors, epidemiology, pathology, and therapeutic approaches for retinoblastoma. We specifically focus on the genetic and molecular characteristics of retinoblastoma, including mutations that cause key signaling pathways involved in the DNA repair, cellular plasticity, and cell proliferation to become dysregulated.

## 1. Introduction

**Figure 1:**
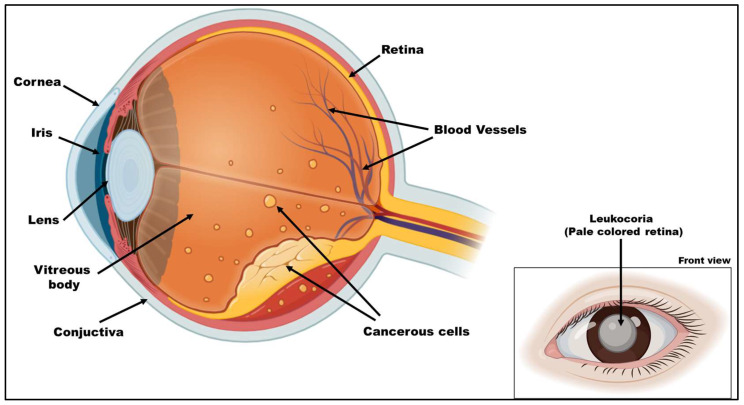
Anatomy of the eye showing the development of retinoblastoma. The illustration depicts a cross-sectional view of the human eye highlighting the presence of cancerous cells within the retina, indicative of retinoblastoma. The tumor cells originate in the retinal layer and are shown spreading toward the inner layers of the eye. The inset image displays a clinical manifestation of retinoblastoma, often seen as a white reflex (leukocoria) in the pupil. The figure was created in Biorender by V.Y. (2025) https://app.biorender.com/illustrations/6893d3bab24c555bb9767535 (accessed on 14 September 2025).

RB is the most common malignant intraocular tumor in children, accounting for about 8000 new cases diagnosed globally each year. Prior research has demonstrated notable variations in the prevalence of RB according to factors such as gender, ethnicity, and infections brought on by inadequate sanitation [1,2]. However, the most recent research downplays the importance of these variations and believes that RB incidence is similar worldwide. Even though RB is rare, scientists are interested in it since the RB1 is the first known tumor suppressor gene. There are 23 pairs of chromosomes in human cells, including the sex chromosomes. Half of these chromosomes come from the mother, and the other half come from the father. Every chromosome has a protector gene called a cancerous growth inhibitor protein. In RB, a specific tumor suppressor RB1 functions to inhibit the development of the cancer. However, genetic mutations or alterations in this gene lead to the formation of RB in infants. These genetic changes in retinal cells disrupt normal growth regulation, resulting in tumor development [1]. Retinal cancer is a treatable malignancy that can lead to retinal viability following early therapy. However, if left untreated, it is always fatal. Delayed treatment can lead to advanced tumors, which can impair vision and increase the risk of metastasis [2]. As shown in Figure 1, the most common symptoms of RB are leukocoria and strabismus. In 60% of instances, the presenting symptom is leukocoria. The early sign of this is a change in the red reflex, which often goes unnoticed, leading to a diagnosis at a later stage, associated with poor prognosis. Strabismus, which is frequently linked to retinal cancer, is another common initial indication for retinal tumors. Less common clinical manifestations may be observed, typically indicating advanced stages of the disease [3,4].

## 2. Types of Retinoblastoma

### 2.1. Hereditary Retinoblastoma

**Figure 2:**
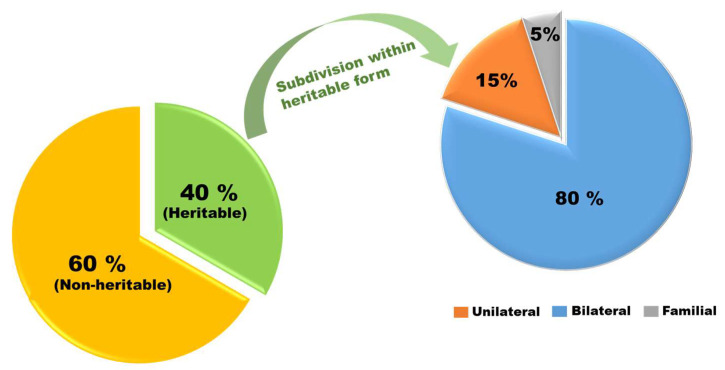
Distribution of sporadic and hereditary forms of retinoblastoma. The left pie chart illustrates that approximately 60% of RB cases are non-heritable, while 40% are heritable. The right pie chart provides a subdivision of the heritable cases, showing that approximately 80% present as bilateral disease and approximately 15–20% as unilateral disease. Among all heritable cases, positive familial history ranges from approximately 5–15%, with the remainder occurring sporadically due to de novo germline mutations.

The normal RB1 helps regulate uncontrolled cell growth, but genetic mutations can prevent it from functioning properly. Depending on the timing and location of RB1 allele alterations, RB can manifest in two different forms [5]. It might be an inherited gene from one’s parents or it can be a random (sporadic) gene alteration. RB is congenital in one out of three cases of the disease; the altered gene is found in every cell in the body. It is referred to as inherited RB that typically affects both eyes when it manifests in this way. Additionally, it raises the risk of developing uveal melanoma and sarcoma, among other malignancies. Despite being referred to as ‘heritable’ (or ‘hereditary’), the majority of these young patients possess no relatives with a history of malignancy; therefore, the RB1 mutation in the gene does not originate in their parents. The genetic variation in such children begins through embryogenesis and fetal growth. A tiny percentage of children born with this genetic change inherit it through their parents [6]. As shown in Figure 2, hereditary RB accounts for roughly forty percent of all cases, with 80% of these being bilateral (affecting both eyes), 15% unilateral (affecting one eye), and 5% trilateral (involving both eyes and the pineal gland) [7]. Bilateral RB can be inherited and passed down to the next generation.

### 2.2. Non-Heritable Retinoblastoma

Non-heritable retinoblastoma, which accounts for about 60% of cases, arises when both RB1 mutations occur somatically in a single retinal progenitor cell, typically resulting in unifocal, unilateral disease (Figure 2). No germline mutation is present in this type [8]. While biallelic RB1 loss initiates tumor formation, additional mutations (M3 to Mn) are usually required for tumor progression [8]. Compared with children who have the heritable form, non-heritable RB is often diagnosed at an older age, and these children are not at the same elevated risk of developing subsequent non-ocular malignancies as those with congenital (heritable) RB. Heritable retinoblastoma, by contrast, results from a germline RB1 mutation and may occur in familial or sporadic (de novo) forms. Notably, about 90% of all RB cases are sporadic with no family history, and only ~10% are familial. Thus, the absence of family history does not exclude the possibility of a heritable mutation [9].

## 3. Global Burden of Retinoblastoma

The cancer of the retina has become an extremely widespread pediatric ocular malignancy and it is frequently treatable if detected early and treated according to routine protocols. Currently, high-income countries are investing economic resources that are increasing life expectancy and quality of life in patients with RB. However, such results do not apply to healthcare institutions across nations with moderate to low incomes [10]. These nations in Asia and Africa account for a large percentage of RB cases, often presenting with advanced tumors at diagnosis, which leads to poor clinical outcomes [11]. The likelihood of RB varies with population size and the rate of births. Fortunately, national cancer inventories differ in terms of availability as well as capability. Wealthy nations, such as Japan, the United States of America (USA), and some European countries, maintain extensive national retinal cancer registry systems, whereas lower- and middle-income nations in Asia and African lack such systems. According to current estimates, more than 50 % of all reported cases of RB occur in the pacific region of Asia, with Africa accounting for approximately one-quarter. India has the highest incidence worldwide, with about 2000 new cases each year—roughly 50% more than in China (≈1000 cases annually) and nearly six times more than in the USA (≈325 cases annually) [12]. The highest illness load is observed in large populations with elevated birthrates, such as the regions of Asia and Africa. In the nation of Nigeria, for instance, retinal cancer remains an extremely common ocular cancer, as well as one of the top five pediatric cancers. As well as having the highest frequency, these regions also experience the highest mortality rates, with around 40–70% of children diagnosed with retinal tumors dying in Africa and Asian nations, as opposed to 3–5% in the European Union, Canada, and the USA [13]. In high-income countries, nearly every child with retinal cancer survives, but, in economically disadvantaged nations, barely more than half survive three years after diagnosis, provided they are ever treated. Approximately three out of every hundred infants with retinal tumors suffer damage to their left and right eyes. A better knowledge of the initial symptoms of retinal tumors, increased availability of timely evaluation, and the implementation of current standards as well as therapy for young people living in middle- to low-income nations are critical to improving retinal tumor outcomes universally [12]. Chantada et al. demonstrated a strong correlation between national economic status and survival outcomes in RB, highlighting marked discrepancies across countries [14].

## 4. Epidemiology of Retinoblastoma

RB represents three percent of all tumors. The incidence of cancer of the retina occurs in approximately 1 in 14,000 to 1 in 20,000 live births. In the USA, 300 new instances are reported per year. RB affects both genders equally. Approximately 90% of instances occur before the age of three years. The disease’s incidence varies by geographical area. According to research, the incidence of RB is six cases per million people in Mexico, compared with four cases per million in the USA. The highest incidences are observed in African countries and India [15]. RB occurrence varies not only among different nations but also within regions of the same country [16]. For instance, in some regions, the incidence reaches 12.9 cases per 1,000,000 children under five years of age. The frequency of 0–4 years over the USA has been unchanged in the past few decades, though it has risen among the indigenous black and white Hispanic populations [17]. In a recent study conducted at the tertiary care center in Karnataka, India, a total of fifty-three individuals (twenty-one combined cases) were diagnosed with RB. The study group had a 1.2:1 male predominance. In 21 cases, bilateral RB was identified. White reflex was the most prevalent initial symptom, occurring in 43 eyes (58%) [18]. Proptosis (bulging of the eyeball) was observed in ten eyeballs (13.5%). Seven eyes (9.5%) showed paralytic squint. Two children presented with signs of elevated intracranial pressure, and one developed status epilepticus. Another patient displayed hemochorial with hyphemia. Overall, the majority of the children had advanced illnesses when they first arrived. When comparing children with high-risk characteristics to those without, clinicians found that the former had a notable presentation delay. Just 39.6% of the patients who received treatment at the center finished the prescribed course of treatment. Consequently, it was not possible to conduct an outcome analysis. Nevertheless, the findings indicate that delayed diagnosis and the high frequency of advanced disease remain significant challenges in developing countries. Periodic ocular examinations during routine vaccination visits may facilitate earlier diagnosis and timely referral for affected children [18].

## 5. Intraocular to Extraocular Metastasis: A Major Clinical Challenge in Retinoblastoma Management

**Figure 3:**
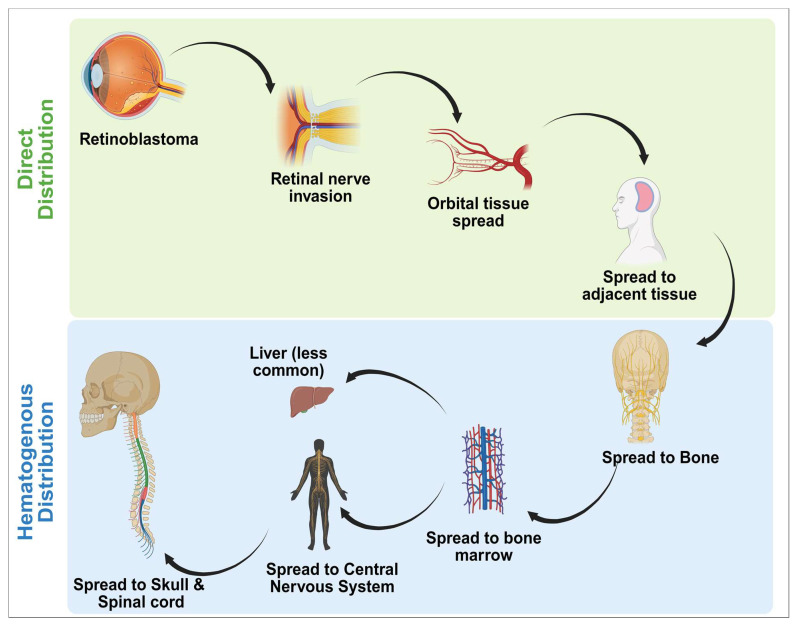
Flow chart representing the metastatic spread of retinal cancer. The figure was created in Biorender by V.Y. (2025) https://app.biorender.com/illustrations/6893c2752447edbbd6fc8384 (accessed on 14 September 2025).

Metastatic illness occurs in approximately 10–15% of individuals with retinoblastoma and is usually associated with particular ophthalmic pathologic characteristics including profound choroidal or scleral encroachment and the invasion of the ciliary system and retinal nerve outside the cribrosa layer. In the future, biological markers detected in plasma will certainly be used to identify individuals at elevated risk of acquiring tiny metastases. One promising approach is the discovery of microRNAs as therapeutic targets [19]. As shown in Figure 3, metastatic dissemination in RB has been observed in the central nervous system, local lymph nodes, cartilage, and liver [20]. Despite advances in retinal cancer therapy, differences in the availability of specialized assistance, as well as in rates of retinal rescue and death, continue to grow [21].

Individuals experiencing retinal cancer in nations with high incomes in North America or Europe face only a three to five percent probability of metastasis-related death, compared to about forty percent in other nations [17]. In contrast, nearly seventy percent of cases occur in countries with limited resources in Africa and Asia. Due to reduced death rates in high-income nations, ocular malignancy counseling now prioritizes globe preservation, ocular rehabilitation, and improved quality of life over metastasis preservations. In contrast, children with retinal cancer in low-resource nations face a much higher risk of both vision loss and death from the disease [22].

**Pathways/Routes of Metastatic Spread**: The cancer of the retina may metastasize to the eye orbit, the nerve that supplies vision to the central nervous system, or the space under the cerebellum, leading to leptomeningeal dissemination. It may additionally undergo hematogenous metastasis into the bones, bone marrow, or hepatic. Occasionally, cancer extends to the surrounding lymphatic systems, as shown in Figure 3 [23].
**Direct Distribution**: Retinal nerve—the remarkably typical route is via the nerve that supplies the vision, particularly if the tumor extends above the layer of lamina cribrosa. It may result in the engagement of the central nervous system (CNS), involving the cerebral cortex.
**Orbit Tissue**: Tumors migration to the orbital cavity may culminate in proptosis (eye bulging) along with additional localized indications.
**Adjacent tissues:** The tumor may also spread to neighboring retinal and periocular tissues, especially the layer of sclera or external muscles of the eye [24].
**Homogenous Distribution**: Circulatory system—tumor cells may penetrate the bloodstream and propagate through various regions. Widespread hemorrhage is frequently caused by systemic complications, often originating in the bone marrow or organs such as the liver.
**Lymphatic Distribution**: The cancer of the retina may expand to localized lymph nodes, especially those in the preauricular, cervix, or submandibular regions. The treatment method mentioned above is no longer standard but retains some historical importance.
**Common sites of Metastases**: Bone and bone marrow (musculoskeletal metastasis): metastasis to bone is primarily localized to the head, lengthy bones (including the thigh bone and elbow), or hips. Such tumors may result in discomfort, edema, or degenerative cracks. Invasion to bone marrow can cause pancytopenia (a drop in red and white blood cells or thrombocytopenia), leading to anemia, a greater vulnerability to illnesses, or symptoms of bleeding.
  Liver (pulmonary metastatic lesions): symptoms include hepatomegaly (expanded hepatic), yellowing of the skin, and stomach discomfort.
  Central nervous system (skull and spinal cord): acute elongation through the nerve that supplies vision may result in cerebral dissemination. Consequences could involve migraines, convulsions, and disorders of the brain [25].

## 6. Risk Factors of Retinoblastoma

Currently, the sole known cause of RB is a genetic mutation or gene alteration. The hereditary variant of RB accounts for around 40% of occurrences and invariably affects extremely young children, usually one year old or less. When RB affects both eyes, it is almost always due to a heritable germline RB1 mutation. However, only 10–15% of children with RB have a family history of the condition, since many germline mutations arise de novo. Although less common, heritable RB can sometimes present in only one eye. Importantly, both heritable and non-heritable forms of RB are caused by genetic mutations in the *RB1* gene. Children with bilateral RB or the genetic form of unilateral RB are more likely to acquire additional cancers. Research suggests that the recognized risk factor for RB, other than inheriting the mutation, is young age. A large proportion of children diagnosed with RB are under five years of age, with many presenting as infants. This increased susceptibility in younger children is due to the abundance of retinal precursor cells (retinoblasts), which are the targets for RB1 mutations. People of all races develop RB at similar rates, while those in impoverished neighborhoods have poorer disease outcomes because of limited healthcare access [26].

In some studies, the only characteristics consistently identified as high-risk factors for recurrence throughout cancer organizations were post-laminar retinal canal intrusion, involvement of retinal neuron layers, and extra-skeletal tumor progression [27]. On the other hand, RB is the consequence of a complex combination of several variables, including young age, family history, parental residency, mother feeding pets during pregnancy, and father was subjected to dangerous pollutants six months before conception [28]. Early identification of risk variables, along with prompt medical attention, may reduce the incidence of recurrence and mortality. In high-income countries, malignant retinal tumors are estimated to affect fewer than 10 percent of patients. High-risk RB causes metastases in 24% of individuals who do not get systemic chemotherapy, compared to about 4% of those who do. The term “high-risk retinoblastoma” refers to cases with a markedly elevated probability of acquiring this malignant ocular neoplasm, most often due to hereditary genetic abnormalities. The principal risk factor is the existence of a germline mutation in the *RB1* gene, which may be inherited from an affected parent (heritable form). High-risk children are those with a known germline RB1 mutation or a positive family history of retinoblastoma, as they have an increased likelihood of developing the disease. Bilateral retinoblastoma represents a clinical manifestation of this heritable form, rather than a risk factor. These cases are also associated with an increased risk of trilateral retinoblastoma, which typically presents as bilateral ocular tumors together with a pineal gland tumor, but in some patients may involve other midline intracranial neuroectodermal sites. Additionally, these people are more likely to develop secondary cancers, particularly if they receive radiation treatment. However, it makes a considerable contribution to RB-related mortality in underdeveloped countries [29].

## 7. Genetics of Retinoblastoma

RB1 stretches 180 kb and contains 27 exons. It is found on chromosome 13q14.2 [30]. The gene, also known as RB Transcriptional Corepressor 1, codes for a 4.7 kb mRNA transcript. More specifically, the databases Ensemble and Gene cards show that the gene extends between chromosome 13:48,303,744 through 48,599,436 (GRCh38:CM000675.2) (GRCh38/hg38), encompassing 295,693 nucleotides and containing a fundamental regulator [31]. The RB1 gene shows a wide spectrum of pathogenic variants, with more than 500 distinct somatic and germline mutations identified to date that can give rise to retinoblastoma [32]. It is known that exons 1–25 contain the bulk of pathogenic point mutations. Eighty-five percent of these mutations are insertions/deletions or single-nucleotide variants (SNVs) located within the 180 kb RB1 coding region. The remaining alterations are attributed to mutations in the promoter and intronic regions [33,34]. Numerous contemporary high-throughput approaches have recently been used to discover new infectious variations of the RB1 protein. All of these mutations result in a premature stop codon, and many frameshift insertions or deletions likewise lead to truncated RB1 protein products through the generation of premature stop codons. Exon skipping outside of the frame as a result of splice site variations is also frequently observed, leading to shortened proteins. Additionally, splice donor site alterations at the intron 12 GT consensus sequence of RB1 have been documented. It has also been observed that *RB1* gene functionality can be disrupted by intronic mutations that affect splicing or regulatory sequences. In addition, highly polymorphic markers such as the RB1.20 microsatellite and minisatellites have been identified within the gene and are widely used in linkage and genetic analyses [35].

## 8. Epigenetic Mechanisms of Retinoblastoma

In addition to the non-coding RNAs mentioned previously, it has been discovered that RB etiology is also caused by other epigenetic processes. The global methylation patterns of RB cells have revealed new information on the need for epigenetic modifications to support the growth and progression of RB [36]. Research has demonstrated that, in addition to RB1 inactivation, RB creation and progression depend on epigenetic dysregulation of tumor-promoting pathways. This suggests that the epigenetic blueprint may function as a biological biomarker for prediction. However, the predictive significance of DNA methylation patterns in RB has only recently been suggested in the last 8 years [37]. The promoters of numerous important genes exhibit aberrant methylation, which leads to changes associated with RB. RB1 was among the first to be examined for DNA methylation. The important promoter region of the *RB1* gene, which is typically unmethylated, is encompassed by a CpG island located upstream of exon 1. A known factor that causes sporadic RB is hypermethylation of the RB1 promoter DNA, which is correlated with decreased pRB1 protein expression [38]. In 13 percent of sporadic unilateral cases, the RB1 is hypermethylated, and a paternal allele was found to be specifically methylated [39]. A study by Li et al. indicated the use of genome-wide DNA methylation profiling to determine the level of methylation of tumors including RB [40]. They discovered 294 genes in which DNA methylation at the promoter or within the gene body was directly associated with regulation of gene expression. Investigations into RB have also considered additional genes’ methylation states. Markovic et al. have reviewed more such studies, which demonstrated that several genes have abnormal promoter methylation in RB, including MGMT, RASSF1A, CASP8, MLH1, CDKN2A/p16INK4A, etc. [41].

## 9. Non-Heritable Causes of Retinoblastoma

In approximately 60% of cases, RB arises from non-heritable, somatic mutations in the *RB1* gene within retinal cells. In such cases, individuals with two normal copies of RB1 never develop retinoblastoma unless both alleles undergo somatic mutation in a retinal cell. These differences in the genetic origin of RB account for the varying risks of tumor development among affected individuals [9]. In most cases, one copy of the RB1 has undergone a heritable mutation (germ cell mutation), present in all of a child’s cells. This means the child has a fifty percent potential of passing the altered gene to their children. This is called an autosomal-dominant type of distribution. A small proportion of children with RB carry a germline mutation in one RB1 allele present in all their cells. Tumor formation occurs when the second RB1 allele is inactivated in retinal cells. In most of these cases, both parents have normal RB1 alleles. The child’s mutations develop after fertilization, but well before tumor formation. This is called the de novo germline mutation and is a one-off event on the scale of the entire population. Most cancers are driven by genetic and epigenetic alterations that disrupt the normal control of cell growth, division, and survival. These alterations may be inherited (germline mutations passed from a parent) or acquired (somatic mutations that arise after conception, either during embryonic development or later in life). RB most commonly (about 60% of cases) arises from non-heritable, somatic mutations in the *RB1* gene, which are not passed on to offspring. The remaining cases are heritable, caused by a germline RB1 mutation inherited from a parent or occurring de novo.

## 10. Symptoms and Diagnosis of Retinoblastoma

A lumbar puncture with a spinal tap may be performed at the time of systemic staging, though many institutions do not include this exam in the workup of a newly diagnosed patient. If imaging exams are inclusive of disease outside the eye, additional tests may be performed to stage both the ocular and extraocular disease. This may include a bone marrow aspiration, bilateral bone marrow biopsy, or magnetic resonance imaging (MRI) of the human brain and orbits [42]. An ultrasound or computed tomography (CT) scan can be conducted to check the presence of calcifications in the tumors. Since RB may spread to the brain, spinal cord, or other parts of the body, children with RB undergo systemic imaging to detect the extraocular disease. Infants younger than three months of age often required examination under anesthesia for proper evaluation of the eyes as they cannot cooperate with indirect ophthalmoscopy.

In addition to clinical and imaging studies, molecular genetic testing of the RB1 gene plays an essential role in diagnosis and management. Genetic analysis identifies children with heritable RB who may transmit the mutation to their offspring, as well as unilateral patients at risk of bilateralization. It also allows the detection of non-carriers within families, sparing them from repeated surveillance and procedures requiring anesthesia. Thus, genetic testing complements ocular and systemic examinations, guiding both treatment planning and family counseling.

**Figure 4:**
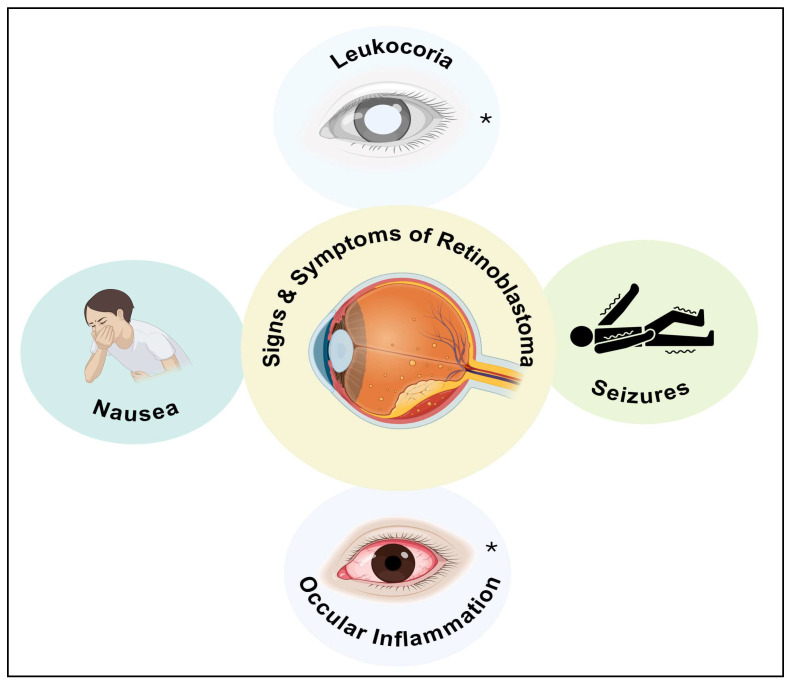
Schematic representation of signs and symptoms of retinoblastoma. * indicates symptoms that occur only in children and are eye related, while the other symptoms apply to adults. The figure was created in Biorender. V.Y. (2025) https://app.biorender.com/illustrations/6893d3bab24c555bb9767535 (accessed on 14 September 2025).

Retinal cancer is the most common recurrent ocular neoplasm in children and is diagnosed by an ophthalmologist, preferably a pediatric ophthalmologist, after a complete examination of the retina with indirect ophthalmoscopy. Symptoms of RB, as shown in Figure 4, include leukocoria, which is the whitening of the pupil of the affected eye, often visible in flash photography. The discoloration occurs because the tumor obstructs the passage of light through the lens and onto the retina. Other presenting features include strabismus, redness, or scleral vascular congestion. Less common symptoms include visual impairment, hyphema, proptosis, and photophobia. A 2015 study reported that leukocoria and strabismus were the most frequent presenting signs in both RB and pseudo-RB. In conclusion, inaccurate referral diagnosis remains a significant challenge for ocular oncology facilities. Proper management of pseudo-RB and RB cases, as well as avoiding unnecessary diagnostic or therapeutic operations, depends on a thorough evaluation and precise diagnosis [42].

## 11. Multidisciplinary Treatment Strategies for RB

**Figure 5:**
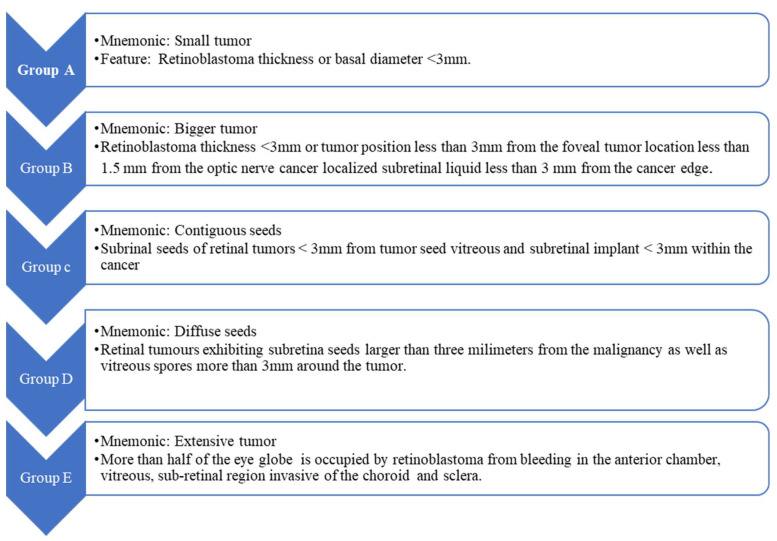
International classification of the pathophysiology of retinoblastoma and chemotherapy options for treating this pediatric disease.

Treatment for RB remains individualized and depends on multiple factors, including family psychosocial issues, cultural attitudes, germline mutation status, accessible institutional resources, and the staging as classified by the International Classification of Retinoblastoma (ICRB), as shown in Figure 5 [43]. RB specialists generally have the same fundamental objectives: preservation of the globe (saving the eye), prevention of metastatic spread, optimization of vision, and saving the patient’s life. When the illness is identified in the limited ophthalmic phase, the currently used therapies maintain good lifetime percentages; however, newer drugs have focused on additional development in worldwide retention and offering the best achievable eyesight outcome. In areas where a wide range of remedies are available, the advancement of these restorative methods has led to formerly unattainable rates of recovery and global salvaging.

### 11.1. Primary Diagnosis and Subsequent Assessment of RB

It is important to ascertain the size of the tumor, both inside and outside the eye, before making treatment plans. This assessment helps to determine disease severity and whether the malignancy has spread beyond the globe. Each affected eye is then assigned a cancer stage [44]. Clinical examination under anesthesia, ultrasound, and MRI are used to assess tumor dimensions, with special attention to the tumor’s connection to the optic nerve. A pineoblastoma, which manifests as trilateral RB, can also be evaluated using brain MRI. Because radiation exposure may raise the chance of developing malignant neoplasms in the future, a CT examination is not advised. To decide on the best course of treatment for the child if retinal cancer has progressed beyond the eye, the cancer’s phase can be diagnosed. RB can be tiny and found using OCT within individuals with relatives with a history of retinal tumors, as well as in rare cases where the child has strabismus or poor eyesight [45]. Making medical and personal decisions about treatment can be facilitated by accounting via a qualified medical geneticist, licensed biological therapist, or certified specialized biological nursing staff to educate affected people, as well as their loved ones regarding the characteristics, manner of transmission, and effects associated with retinal tumors.

### 11.2. Intravenous Chemotherapy

Systemic IVC became available in the first half of the 1990s and is now a vital therapy option for retinal cancer. IVC generally entails a monthly insertion of two, three, or four chemotherapeutic drugs utilizing a centralized and periphery catheterization for a total of six to nine cycles. The most commonly prescribed chemotherapy regimen consists of three medications: vincristine, etoposide, and carboplatin (VEC) [46]. When neurotoxicity is a concern, pediatric oncologists in Monterrey, Mexico, occasionally switch from vincristine to cyclophosphamide; nevertheless, the first group of medications is inclined to cause myelitis or recurrent cystitis [47]. When administered within 48 h of the thermal disturbance, cryotherapy immediately before chemotherapeutic was shown to improve medication access to the ophthalmic locations [48]. Patients with high-risk features, such as bilateral illness, proven mutations, a family history of retinal tumors, optic nerve damage or choroidal invasion, are often treated with IVC chemotherapy, which has a protective effect against metastases and long-term second malignancies [49].

### 11.3. External Beam Radiotherapy

Globe salvage therapy was performed with external beam radiation (EBRT) before IVC was introduced. Since an effective treatment for RB was introduced, EBRT has largely lost its historical significance in the majority of developed nations because of the numerous adverse effects it has and the improved results that followed. Nonetheless, EBRT continues to play a part in cases of orbital recurrence and extension of extraocular tumors, with positive optic nerve border after enucleation. It has been found that 71% of patients with orbital RB respond well to the mixture of EBRT plus IVC for tumor management [50]. Tear deficit, eye dryness, filamentary keratopathy, cataract, irradiation retinal degeneration, optical nerve damage, and/or ocular development retardation leading to cosmetic deformities are among the radiation adverse consequences linked to EBRT [51].

### 11.4. Plaque Radiotherapy

Plaque radiation, also known as brachytherapy, was first introduced in 1929 and was a means of salvage management for recurring tumors after EBRT. Nowadays, when chemo-resistant small-to-medium tumors (≤16 mm in greatest receptive size or >3 to <9 mm in width) relapse after IVC and IAC, brachytherapy is usually employed as a subsequent treatment [52]. This can include localized vitreous and subretinal seeding. In the absence of choroidal and macular tumors, generalized front region retinal tumors, regardless of IVC, can also be treated with plaque irradiation [53]. For the best tumor coverage, the greatest basal diameter is usually increased by 2 mm as a safety margin.

### 11.5. Enucleation

Despite significant advances in globe-salvaging techniques, eye-globe enucleation remains a crucial treatment for advanced RB. In most group D and particularly group E cases (grouping based on IIRC classification), where the disease is life threatening, enucleation is often necessary to save the patient’s life. It is especially indicated for tumors with extraocular extension, optic nerve or choroidal invasion, or when the disease has proven resistant to conservative therapies [54]. Enucleation is the surgical extraction of the whole eye globe and severance from all orbital acquaintances, including the transection of the retinal nerve. In late intraocular illness, conservative treatments such as systemic chemotherapy, intra-arterial chemotherapy, and targeted therapies (such as laser or cryotherapy) are less successful, even if they have increased eye preservation rates, which can reach up to 70–85% in early-stage RB. Enucleation provides the best safety margin in these situations, successfully halting metastases and extraocular dissemination. It performs a crucial life-saving role even when the affected eye is sacrificed, particularly when the eye preservation rate is less than 20% because of complications such as neovascular glaucoma or substantial tumor involvement. Given that the earliest records of it date to 2600 BC, it is among the most ancient ophthalmic operations [53].

### 11.6. Genetic Counseling

Chromosomal analysis and molecular genetic testing can help determine whether an individual carries a germline RB1 mutation. This allows for the accurate identification of first-degree relatives who are mutation carriers, who have approximately a 90% likelihood of developing RB, distinguishing them from non-carriers who are not at increased risk. Genetic testing therefore provides a more precise estimate of risk than family history alone. In addition, patients with heritable RB have an increased lifetime risk of developing subsequent primary non-ocular malignancies, making genetic counseling essential for long-term management and family planning. Screening for RB1 mutations is clinically important for distinguishing heritable cases from non-heritable cases. Tumor analysis, in combination with blood testing, can establish whether the mutation is present in the germline (heritable) or restricted to tumor tissue alone (non-heritable). This information is critical for guiding clinical management, family counseling, and avoiding unnecessary procedures in non-carriers, while also contributing to research on the long-term impact of RB1 alterations.

Candidates for genetic counseling include individuals with a track record of bilateral RB, as well as those with unilateral disease and the relatives of affected individuals. Genetic counseling is also essential for planning surveillance and management strategies in RB-prone families. In addition, unilateral RB survivors should undergo long-term ophthalmic monitoring, including visual function assessment, since they are at risk of developing hereditary RB in the other eye.

### 11.7. Long-Term Follow-Up and Monitoring

The life expectancy of survivors of RB has generally improved over time, as contemporary therapies confer fewer treatment-related sequelae. Given the value of patient medical data, the demographic details and future vision results of survivors are important. Some contemporary medical facilities have reported on their survivors and their outcomes, characterizing the different disease manifestations in children and adults with hereditary RB. However, the comparatively rare prevalence of hereditary RB means that very few RB specialists have patients solidly into their adulthood or even older adults/survivors; there have, until now, been international attempts to analyze this unique cohort’s visual function and overall health. Significant unmet needs exist for advancing methodologies in both occupational and global quality-of-life assessments conducted on a population-based sample of chronic RB victims.

Before completing treatment, all patients need to undergo the following evaluations: a vision assessment; examination under anesthetic (or examination of an under-anesthetic depending on whether the patient will have further examinations); examination by a member of the multidisciplinary treatment team; radiation retinopathy and RB recurrence through serial imaging; the timing of specialist follow-up; and long-term oncology/general practitioner follow-up. Expectation management as to visual outcomes post-treatment and post-remission is important, as are short discussions of current long-term radiation retinopathy and second primary malignancy data. Survivors of hereditary RB are a relatively unique cohort in terms of their ongoing cancer risks and require consistent coordinated follow-up from dedicated RB specialists. Many quality-of-life studies show vision is associated with daily functioning and mental health, and visual problems have been associated with neuro-psychological, economic, educational, and social sequelae. The visual development of children with hereditary RB may not necessitate a significant discrepancy with either their healthy peers or their parents with hereditary RB. Through premature apoptosis of susceptible developing retinal cells and peri-ocular adnexa, other difficulties are theoretically superior in sporadic RB, but further specific hereditary group comparisons are needed.

## 12. Clinical Trials Investigating Novel Therapeutic Approaches

T-cell immunotherapy has demonstrated remarkable anticancer results in severe B-cell malignancies and non-Hodgkin’s lymphoma. This treatment involves isolating the patient’s T cells and modifying them to express CD19-specific receptors for chimeric antigens (CARs) [55]. In human RB cell lines, CD171 and GD2 have been identified as viable targets for in vitro testing, with CAR T-cell therapy demonstrating strong antitumor potential against RB. In one of the two-orthotopic RB models, Wang et al. published a CAR design, which comprises the T-cell antigen receptor CD3 chain, the end domain associated with the co-receptor CD28; this is a single-chain changeable segment generated via the monoclonal antigen dinutuximab [56,57]. The researchers demonstrated that subretinal administration of CAR T-cells inside a hybrid gel inhibits RB in susceptible rodents, without any apparent impairment occurring to the adjacent retina; moreover, IL-15 promotes the expansion and stability of GD2 CAR T-cell receptors [56,57]. Due to accessibility, the eye is a good candidate for topical administration of CAR T-lymphocytes. Additionally, ophthalmic distribution may result in allogeneic drugs being rejected, making it more feasible to administer them at other locations.

Oncolytic virotherapy involves genetically modified or attenuated viruses, such as VCN-01, which selectively infects tumor cells, replicates within them, and induces systemic immune reactions [57]. VCN-01 is an engineered oncolytic adenovirus designed to replicate only in tumor cells with high levels of free E2F1, which is a symptom of a malfunctioning RB. In vitro, VCN-01 has been successfully shown to eradicate patient-derived RBs in tumor models. In mice with RB xenografts, IvitC injection of VCN-01 promoted tumor apoptosis, preserved retinal longevity, and inhibited micro metastatic spread to the cerebral cortex, despite causing some ocular inflammation. A first in-human clinical trial is currently underway at the SJD Barcelona Children’s Hospital to evaluate the safety and efficacy of VCN-01, particularly in RB cases that are resistant to conventional chemotherapeutic treatments [58].

## 13. Nanoparticle-Based Therapy for Retinoblastoma

Nanoparticles have been widely studied in relation to the detection and management of eye disorders. Their micro dimensions, form, and exterior characteristics allow them to penetrate visual organs, eliminating visual obstacles and increasing accessibility or effectiveness in therapy. They provide the benefits of regulated, enduring, and localized medication administration while limiting adverse reactions [59]. Several investigations have shed light on nanoparticles’ potential as transporters for cancer-fighting drugs in the therapeutic management of cancer. Many such nanomaterials have been authorized, whereas others are undergoing clinical and preclinical investigations across a variety of malignancies, including solid cancers, cancers of the breast and ovary, and lymphoma [60].

### 13.1. Bioactive Nanoparticles

**Figure 6:**
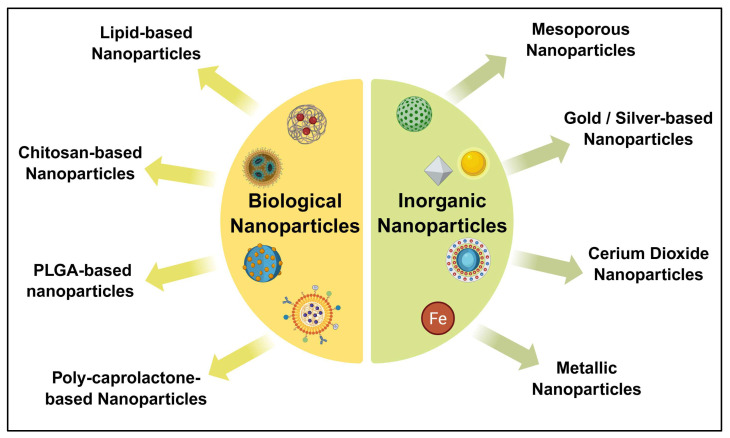
Schematic illustration of nanoparticle-based strategies for retinoblastoma. Bioactive nanoparticles consisting of lipids and polymers. Inorganic nanoparticles consisting of metallic & rare-earth metallic compounds. The figure was created in Biorender. V.Y. (2025) https://app.biorender.com/illustrations/68bff5f9855817ad1ab8b08d (accessed on 14 September 2025).

Natural bioactive nanoparticles are molecules of organic materials formed by non-covalent interactions between molecules, making them extremely adaptable and supplying an avenue for expulsion through the body’s tissues, as depicted in Figure 6. The most prevalent kinds of natural tiny particles used in RB rehabilitation are nanoparticles composed of lipids (LNPs), lactic acid nanoparticles, and polymer-based (e.g., poly-caprolactone (PCL), a substance called chitosan (CH), polylactic-co-glycolic acidic substances (PLGA), and PMMA (polymethylmethacrylate)) NPs, which are biocompatible, have elevated absorption rate in RB tissues, and have no apparent contaminants in addition to beneficial photothermal and acoustic imaging attributes in many different ways [61].

### 13.2. Inorganic Nanoparticles

Inorganic nanoparticles, which are made up of non-carbon-based particles, have drawn an enormous amount of study in the optical administration of drugs due to their ability to change their dimensions, shape, and crystallization, as well as their substantial surface area, elevated probability of exterior drug bonding, and ease of integration, as depicted in Figure 6. The most frequently employed nanotechnology for the ingestion of chemotherapy drugs in the management of RB illness involves mesoporous silica, oxide of iron (Fe_2_O_3_), gold (AU), silver (AG), and cerium dioxide NPs [62].

### 13.3. Transport of Lipid-Encapsulated Nanostructures

NLCs are the subsequent version of SLNs with fluid lipids displacing the partial lipid solid elements found in SLNs, resulting in a larger pharmaceutical storage region. NLCs outperform standard transporters for the visual administration of medications in several respects: notably, increased lubricity, the capacity to boost preservation equilibrium, greater absorption, flexibility, extended lifespan, minimal negative reactions, and dependence on tissue distribution [63].

Distribution of medications within the retina is difficult due to safeguards in the ocular structures. Furthermore, the administration of drugs using different nanotechnologies works well sufficiently to overcome these constraints. The most widely utilized and beneficial multi-functionalized tiny particles for curing RB include multi-functionalized NPs, lipid-based NPs, and metal-based NP multi-functionalized nanomaterials for the ophthalmic administration of medications that can overcome optical hurdles and eliminate retinal cancer; however, generated NPs possess the ability to preserve the medicinal molecule and enhance persistence duration. Polymerized NPs are disposable and might be utilized for targeted or reliable intravitreal allocation in RB [64].

### 13.4. Polymerized Nanoparticles

Polymerized nanoparticles (PNPs) number among the most effective nanoparticles with multiple functions for the medical management of a retinal tumor. They have been extensively investigated and have received far greater scrutiny than other particles. As a result, PNPs may be more efficient at identifying and eliminating malignant cells. Polylactic acid nanoparticles containing rhodamine dye have been used in rat models to deliver agents to the retina. During a single intravitreal injection, these nanoparticles were able to diffuse through the layers of the retina and accumulate within the macular pigmented cells for up to four weeks. Fortunately, only minor irritation was observed in the lower vitreous humor and the ciliary body, which subsided within forty-eight hours [65].

### 13.5. Lipid Encapsulated Nanoparticles

Lipidic tiny particles contain molecules of lipids as a molecular framework. Lipid-based tiny carriers are establishing themselves as attractive options for administering medicines for the treatment of specific cells inside the cornea. Due to their biological compatibility and delivery through eye flexibility, small particles of lipids (LNPs) are widely utilized as nucleic acid delivery systems for the treatment of disorders of the eyes because they can cross the optical membrane and effectively transfect DNA throughout different tissues of the eyeball. The dimension of phospholipid tiny carriers influences their capillary digestion, dispersion, and biological distribution, resulting in an appealing substrate and enabling the precise administration of drugs. Membrane-based tiny particles encompass many transportation systems [66].

## 14. Treatment Modalities of Retinoblastoma: Unilateral vs. Bilateral

The main goals in treating retinoblastoma are to save the patient’s life, preserve the eye, and maintain vision, and these objectives influence the choice of treatment strategy. This method was customized for each patient, considering elements such as the tumor affecting one eye or both eyes, the tumor’s position and stage, the possibility of vision loss, and family wishes [67]. A study conducted by Mohammad et al. compared treatment outcomes in 587 patients with unilateral versus bilateral retinoblastoma. In this study, IIRC classification was used to categorize the disease stage. Treatment decisions were made on the basis of vision prognosis, retinoblastoma stage and type, age factor and subject choice. Primary enucleation was typically performed for unilateral cases, while intravenous chemotherapy, systemic chemotherapy, and intravitreal melphalan were used for eyes with persistent vitreous seeds that did not respond adequately to systemic therapy [68]. In bilateral RB, treatment strategies aim first to save the child’s life and, whenever possible, to preserve vision in at least one eye. Typically, the eye with the more advanced tumor is enucleated, while the other eye is managed using conservative approaches such as chemotherapy, focal therapies, or, when necessary, external beam radiation therapy (EBRT). Although EBRT carries an increased risk of secondary malignancies in heritable RB, it may be considered in selected cases when eye preservation cannot be achieved by other means [69].

## 15. Discussion

Retinal tumors, a type of cancerous eye malignancy commonly found in children, have long been linked to the RB1 variant. Recent studies, however, have expanded our understanding by highlighting the significant roles of additional genetic and environmental factors in the progression of retinal tumors. Beyond the RB1 mutation, other gene abnormalities, such as MYCN amplification, have been identified, indicating a more complex genetic basis for the disease. These discoveries open the door to new treatment strategies. Moreover, the field of cancer ecology is gaining attention, with the interactions between retinal tumor cells and their environments, especially immune system cells, being increasingly acknowledged as critical for tumor growth and treatment outcomes. This has led to a reevaluation of chemotherapy’s potential as a therapeutic option. Innovations in scanning and detection are transforming the treatment of retinal tumors. Advanced imaging technologies facilitate easier and more accurate identification, thereby improving prognosis. Additionally, liquid biopsy techniques, which detect tumor DNA in bodily fluids, offer a non-invasive method for detection and monitoring. Personalized healthcare is becoming increasingly prevalent, incorporating treatment strategies tailored to the genetic or genomic attributes of individual tumors. This approach aims to optimize therapeutic benefits while minimizing adverse effects, signifying a marked shift towards customized and effective retinal cancer treatment. These distinctive perspectives highlight the complex characteristics of retinal tumors and advocate for the development of innovative testing and treatment methods, aiming primarily to improve the prognosis for pediatric patients.

## 16. Conclusions

The WHO has designated RB as a priority malignancy in its Global Initiative for Childhood Cancer. Even though it is quite treatable in its early stages, if treatment is not received, it can be lethal. Because of significant advancements in eye-globe salvage techniques over the past few decades, intraocular RB is now the most treatable juvenile malignancy in high-income nations. An unparalleled rate of eye globe and vision preservation has been achieved with the invention of local drug delivery techniques, including OAC and a safety-enhanced IVi injection technique, which optimize chemotherapy exposure in the retinal, subretinal, and vitreous areas. Children with disseminated RB have minimal treatment options, typically restricted to high-dose chemotherapy, stem cell transplant, and local radiation, in contrast to this remarkable improvement in treatment results. Patients with CNS metastases have a considerably worse prognosis since, despite rigorous treatments, they rarely survive. Therefore, results may be enhanced by more recent therapies and better ways of targeting medication delivery to the central nervous system.

The lack of an appropriate molecular targeted therapy for RB, even though it is one of the oldest malignancies to be found, suggests that there are numerous unidentified secondary mutations that contribute to the disease and have yet to be identified. New, more effective treatments with a lower risk of toxicity are required for all cases of intraocular and extraocular illness. Developing new approaches with reduced toxicity is a priority, including immunotherapy, oncolytic virotherapy, and targeted drug delivery systems. Nanotechnology-based platforms may further enhance the precision and safety of these treatments. However, in order to ensure that RB is a pediatric cancer that may be cured, we must advance our knowledge of a number of topics pertaining to tumor biology and efficient treatments.

## Data Availability

Not applicable.

## Abbreviations

The following abbreviations are used in this manuscript:

APC2	Adenomatous Polyposis Coli 2
CAR T	Chimeric Antigen Receptor T-Cell
CGA	Chromogranin A
IIRC	International Intraocular Retinoblastoma Classification
IVC	Intravitreal Chemotherapy
LDHA	Lactate Dehydrogenase A
LNPs	Lipid Nanoparticles
MGMT	O-6-Methylguanine-DNA Methyltransferase
PCL	Polycaprolactone
PLGA	Poly (lactic-co-glycolic acid)
RB	Retinoblastoma
RB1	Retinoblastoma 1 gene
SLNs	Solid Lipid Nanoparticles
SVs	Single Nucleotide Variants
VEC	Vascular Endothelial Cell
